# WeWalk: walking with a buddy after stroke—a pilot study evaluating feasibility and acceptability of a person-centred dyadic behaviour change intervention

**DOI:** 10.1186/s40814-022-01227-5

**Published:** 2023-01-13

**Authors:** Jacqui H. Morris, Linda Irvine, Tricia Tooman, Stephan U. Dombrowski, Brendan McCormack, Frederike Van Wijck, Maggie Lawrence

**Affiliations:** 1 School of Health Sciences, University of Dundee, Dundee, UK; 2 Kinesiology, University of New Brunswick Fredericton, Fredericton, New Brunswick Canada; 3 Nursing and Midwifery, University of Sydney, Sydney, New South Wales Australia; 4 School of Health Sciences Queen Margaret University, Edinburgh, UK; 5 School of Health and Life Sciences, Glasgow Caledonian University, Glasgow, UK

**Keywords:** Stroke, Physical activity, Behaviour change, Feasibility, Walking, Intervention development

## Abstract

**Background:**

Evidence for benefits of physical activity after stroke is unequivocal. However, many people with stroke are inactive, spending > 80% of waking hours sedentary even when they have physical capacity for activity, indicating barriers to physical activity participation that are not physical. WeWalk is a 12-week person-centred dyadic behaviour change intervention in which a person with stroke (PWS) and a walking buddy form a dyad to work together to support the PWS to increase their physical activity by walking outdoors. This pilot study examined the feasibility of recruiting dyads, explored their perceptions of acceptability and their experiences using WeWalk, to identify required refinements before progression to a clinical trial.

**Methods:**

Design: A single-arm observational pilot study with qualitative evaluation.

Intervention: WeWalk involved facilitated face-to-face and telephone sessions with a researcher who was also a behaviour change practitioner, supported by intervention handbooks and diaries, in which dyads agreed walking goals and plans, monitored progress, and developed strategies for maintaining walking.

Evaluation: Descriptive data on recruitment and retention were collected. Interview data were collected through semi-structured interviews and analysed using thematic analysis, guided by a theoretical framework of acceptability.

**Results:**

We recruited 21 dyads comprising community dwelling PWS and their walking buddies. Ten dyads fully completed WeWalk before government-imposed COVID-19 lockdown. Despite lockdown, 18 dyads completed exit interviews. We identified three themes: acceptability evolves with experience, mutuality, and person-centredness with personally relevant tailoring. As dyads recognised how WeWalk components supported walking, perceptions of acceptability grew. Effort receded as goals and enjoyment of walking together were realised. The dyadic structure provided accountability, and participants’ confidence developed as they experienced physical and psychological benefits of walking. WeWalk worked best when dyads exhibited relational connectivity and mutuality in setting and achieving goals. Tailoring intervention components to individual circumstances and values supported dyads in participation and achieving meaningful goals.

**Conclusion:**

Recruiting dyads was feasible and most engaged with WeWalk. Participants viewed the dyadic structure and intervention components as acceptable for promoting outdoor walking and valued the personally tailored nature of WeWalk. Developing buddy support skills and community delivery pathways are required refinements. ISCTRN number: 34488928.

**Supplementary Information:**

The online version contains supplementary material available at 10.1186/s40814-022-01227-5.

## Key messages regarding feasibility

- What uncertainties existed regarding the feasibility? The feasibility of recruiting community-dwelling people with stroke and walking buddies to form dyads for participation in WeWalk was uncertain because this type of intervention had not been previously undertaken with people with stroke. It was unclear whether buddies could be successfully recruited and whether dyads could work together and engage with intervention components.
- What are the key feasibility findings? Recruiting and retaining dyads was feasible until COVID-19 interrupted the study. Recruitment through physiotherapy services was feasible but more participants were recruited through community routes. The intervention was implemented as intended and was valued by participants for its impact on walking and confidence and for the person-centred approach to goal setting. Dyads functioned best when both members were mutually engaged.
- What are the implications of the feasibility findings for the design of the main study? WeWalk shows potential, but more work is necessary to refine recruitment and delivery strategies. Development of tools to evaluate how existing relationships can best support WeWalk, along with training for buddies in support strategies, will be required. Consideration of community recruitment and implementation strategies and appropriate outcome measures for evaluation of effectiveness is required prior to the main effectiveness study.

## Background

Evidence for benefits of physical activity (PA) after stroke is unequivocal. Systematic reviews and randomised controlled trials demonstrate benefits of PA and exercise participation for functioning, gait and balance, cardiovascular fitness, and quality of life [1–3]. PA participation may also lessen the likelihood of recurrent stroke by reducing risk factors such as high blood pressure and cholesterol [4].

Despite these benefits, and clinical guidelines encouraging PA promotion during and after rehabilitation [2, 5, 6], PWS remain inactive, spending > 80% of waking hours sedentary even when they have physical capacity for activity [7]. Qualitative research with PWS suggests complex barriers to PA [8, 9], and even after participation in post-rehabilitation exercise programmes, regular PA is often not maintained [10, 11]. Finding ways to support regular PA participation after stroke is vital [12]. Regular walking can prevent cardiovascular disease in adults but has not been widely explored as a strategy post-stroke [4]. Qualitative studies indicate PWS regard walking as enjoyable, accessible and affordable, suggesting that walking interventions may be acceptable and effective for promoting regular PA, improving functioning and cardiovascular health [8, 13].

Incorporating theoretically informed behaviour change strategies into a walking intervention may promote and maintain PA participation more than instruction alone [14, 15] because these can address social, psychological, motivational and environmental barriers to activity. Despite widespread use in public health development, testing and implementation of behaviour change strategies to promote PA in stoke has been slow [15].

Social support can influence maintenance of PA [16]. Our previous qualitative research showed that carers often adopt a facilitatory role in supporting PWS to be active in their daily lives, so they could participate in shared activities together. In that study, carers adopted a common-sense approach to setting and achieving goals with the PWS, focused on the PWS physical activity goals and desired outcomes [17]. These findings led us to consider that developing a dyadic intervention could be appropriate to support PWS to be more physically active. Dyadic interventions involve two people working together to support behaviour change of one or both dyad members [18]. A systematic review with meta-analyses suggests that dyadic interventions may be more effective in increasing PA than those targeting individuals alone [19]. The review drew on Transactive Goal Theory [20] to provide a theoretical basis for the focus of dyadic goals, with findings indicating that goal-related outcomes were most likely when both partners share the same goal for the target dyad member, rather than each pursuing individual parallel goals [18], a finding reflected in our earlier qualitative study [17]. Thus, the effort of dyad members is on co-ordinating goals for the dyad member with the clinical condition [20]. The same review showed that dyadic interventions targeting clinical populations with disabilities affecting physical activity capability were more effective than those targeting non-clinical populations. The authors reasoned this was because the clinical need for increased activity i enhanced motivation to be active, a factor that is highly relevant after stroke. Thus, there were good theoretical reasons, but no empirical evidence to suggest that a dyadic intervention focused on physical activity of people with stroke may be appropriate to facilitate walking as physical activity in this heterogeneous clinical population.

We designed a person-centred, 12-week dyadic behaviour change intervention called WeWalk to promote outdoor walking as a regular form of PA after stroke. We co-designed WeWalk with PWS, their families and rehabilitation professionals. WeWalk involves a dyad comprised of a PWS and a walking buddy supported by a trained facilitator. People with stroke suggested that we use the term walking buddy to describe the walking companion. The facilitator supports dyads to identify, enact and review meaningful walking goals for PWS, address challenges and plan for long-term maintenance of walking. Detailed intervention development processes are published elsewhere [21]. This study aimed to pilot WeWalk as the final stage of intervention development. We examined the feasibility of recruiting dyads, explored dyads’ perceptions and experiences of WeWalk, assessed acceptability and identified aspects requiring refinement in preparation for effectiveness testing.

## Methods

### Design

A single-arm observational pilot study with qualitative evaluation conducted with people with stroke who were community-dwelling.

### Sampling and recruitment

Adults with stroke who were able to walk, community-dwelling, and could provide informed consent were eligible for inclusion. Potential participants within four Scottish Health Board areas were invited to participate through invitations from community rehabilitation therapists, stroke organisations and exercise classes and via a press release. Participants expressing interest invited someone known to them to act as a ‘walking buddy’. The walking buddy had to be willing to support the PWS and be able to walk with them. The study researcher (LI) visited dyad members to explain WeWalk and answer questions, after which dyad members provided written informed consent. If PWS could not identify a walking buddy, buddies were identified through local volunteer or exercise after stroke organisations. In these cases, the researcher met with the PWS and volunteer buddy in a neutral venue to introduce them, explain WeWalk, establish compatibility and commitment, and define roles and expectations before the intervention was delivered.

### Intervention

WeWalk was underpinned by research into barriers and facilitators to post-stroke PA [22, 23] and structured using the Behaviour Change Wheel [24], the Health Action Process Approach (HAPA) [25], and person-centred principles developed by one of the research team [26]. The target behaviour of WeWalk was specified as outdoor walking [21]. Detailed intervention development processes, underpinning theories and the intervention logic model are published elsewhere [21]. Briefly, the work evolved from formative qualitative research by the team and from systematic reviews of the literature on physical activity promotion after stroke. To develop WeWalk, barriers and facilitators to physical activity after stroke from that research were systematically mapped to the Capability, Opportunity, and Motivation framework the Behaviour Change Wheel, enabling identification of salient behaviour change techniques (BCTs) from Michie’s taxonomy of BCTs [27]. The BCTs relating to Capability, Opportunity and Motivation were then applied to the concepts within the HAPA, to structure the intervention and support participants’ movement through HAPA stages of developing motivation to walk and volition and engagement in walking, whilst addressing emerging challenges. In this way, HAPA provided a framework for delivery of the components of WeWalk. We designed WeWalk as a person-centred intervention, using person-centred principles to guide delivery by the facilitator to ensure WeWalk aligned with participants’ values, and facilitated their engagement in walking activities in ways that were meaningful to them and their context [21]. The initial intervention was developed and refined through consultation with PWS, their family members and health professionals, before review by experts in behaviour change.

The study researcher (LI), a female public health researcher supported design and delivery of WeWalk whilst employed as the study research fellow. She has 30 years of research experience in designing and delivering complex behaviour change interventions for parents who smoke and to different social groups of people who are regular binge drinkers. She has created handbooks for delivering interventions and has trained lay people to deliver interventions. We decided it was most appropriate to employ a researcher to deliver the intervention rather than health practitioners, so we could gather crucial fieldnote data to inform intervention refinement. Her role involved collecting fieldnote data to explore and refine intervention delivery; information on enactment and how dyads worked, and support required for training of facilitators to inform future delivery.

At an initial face-to-face visit to the participants’ homes, dyad members provided written informed consent, the researcher explained dyadic working and baseline data were collected. The participants were provided with handbooks to explain the intervention and the role of each dyad member—one designed for buddies and one for PWS, and a diary to record and monitor progress. Both the PWS and the buddy were offered a pedometer; however, some participants, who already used recording devices (smart watches, phone apps), did not use pedometers. The researcher facilitated identification of person-centred goals in discussion with the dyad. This was done by discussing how to increase the amount of outdoor walking, undertaken by the PWS. The facilitator asked the dyad how much the PWS walked outside and then the dyad would discuss how this could be increased. The baseline walking of each PWS varied greatly, so goals set were relevant to their baseline. WeWalk was delivered over two further visits and two phone calls. Sessions lasted between 45 and 60 min and involved discussion with the dyad about developing goals together, planning how the goals would be enacted, identifying problems and discussing solutions. The dyad was asked to write down what the walking goal for the PWS would be every week. This was usually an increase in walking, but would be adjusted, depending on how well goals were being attained. The dyad was then asked to make a plan and write down in the diary: when I will walk, where I will walk, how much walking I will do (distance, steps taken; or time to be spent walking) and how my buddy will support me. Every session was audio recorded to assess fidelity of delivery and for refining intervention content and delivery methods for future studies. A final visit at week 12 was conducted to support maintenance and collect interview data. Intervention components and behaviour change techniques are presented in Table 1.

**Table 1:** Outline of the components of WeWalk and associated behaviour change techniques

Intervention component/HAPA constructs	Description	Behaviour Change Techniques [27] *denotes core BCTs
***Discuss benefits of walking with dyad***	Increase motivation to walk by discussing benefits of walking after stroke for physical and mental health and maintaining and improving walking and recovery. Discuss barriers and how others have addressed them by drawing on positive experiences of other PWS. Introduce handbooks	3.1.Social support (unspecified)* 5.1.Information about health consequences 5.2.Salience of consequences 5.3.Information about social and environmental consequences 5.6.Information about emotional consequences 11.2.Reduce negative emotions 15.1.Verbal persuasion about capability
***Discuss working as a dyad***	Explain dyadic working. Discuss how the dyad can work together to develop goals and plans to support walking. Discuss role of walking buddy and benefits of social support for walking. Discuss potential benefits for buddy	1.9.Commitment 3.1.Social support (unspecified)* 12.2. Restructuring the social environment
***Identify and set personally meaningful walking goals***	Explore and develop intentions to walk more. Encourage the dyad to agree personally meaningful walking goals for the PWS, based on the capabilities and desires of PWS. Agree how buddy will provide support (the buddy may not always walk with the PWS)	1.1.Goal setting (behaviour)* 1.3. Goal setting (outcome)* 3.1.Social support (unspecified)*
***Action Planning***	Suggest that the dyad makes an action plan after they have set a goal. Explain that when a meaningful, shared goal based on step counts, time spent walking and on desired activity or walking route has been agreed, laying out a plan provides a way to help achieve their goal. Plan when, where, and how much they will walk for the week ahead	1.4.Action planning* 3.1.Social support (unspecified)* 7.1.Prompts/cues 8.1Behavioral practice/ rehearsal
***Monitor progress, coping planning***	Identify and agree a self-monitoring strategy. Select personally relevant monitoring approach: step-count using pedometer provided or monitor time spent walking, distance, places walked to. Provide pedometers. Discuss completion of activity diary. Anticipate challenges and discuss ways the dyad can negotiate ways to overcome them when they arise	1.2 Problem-solving* 2.3.Self-monitoring of behaviour* 2.4.Self-monitoring of outcomes of behaviour* 3.1.Social support (unspecified)* 4.1Instruction on how to perform the behaviour
***Reviewing goals, problem-solving and maintenance***	Review goals with the dyad, review diaries and progress made towards goals. Review action plans, discuss lessons learned, offer encouragement. Discuss goal revision and review how the dyad is working. Explore challenges encountered, setbacks. Discuss coping planning and discuss building support and reminders to include walking into everyday life	1.2.Problem solving* 1.5.Review behaviour goal(s)* 1.6.Discrepancy between current behaviour and goal 1.7.Review outcome goal(s)* 2.2. Feedback on behaviour 2.7.Feedback on outcome(s) of behaviour 3.1.Social support (unspecified)* 7.1.Prompts/cues 8.3 Habit formation 13.5.Identity associated with changed behaviour 15.1.Verbal persuasion about capability 15.3. Focus on past success

### Data collection

At baseline, we collected demographic data about PWS, buddies and their relationships (e.g. spouse, partner, friend). At study visit one, PWS completed questionnaires evaluating walking and functional mobility. We examined 7-day PA levels using the Physical Activity Scale for the Elderly [28], functional mobility using the Rivermead Mobility Index [29], self-reported confidence in balance using the Activities-Specific Balance Confidence Scale [30], confidence in walking using the Self-Efficacy Questionnaire for Walking [31], and fatigue, using the Fatigue Assessment Scale [32]. Additional File 1 (Supplementary Materials) explains measures and scoring systems. Questionnaires were completed at baseline only to characterise participants as we did not aim to assess effectiveness. All intervention sessions were audio-recorded to generate detailed fieldnotes documenting delivery fidelity and participant responses. Diaries were returned at the final study session to review and assess intervention engagement.

The researcher (LI) conducted in-depth semi-structured interviews in person or by telephone with dyads at the end of WeWalk or at discontinuation due to COVID-19. Interviews lasted between 30 min and 1 h. Because the nature of the study was to identify and address solutions to pragmatic problems, we adopted pragmatism as our methodological orientation [33]. A semi-structured interview guide (Additional File 2) facilitated the exploration of dyadic working, views about intervention components, form of delivery, intervention acceptability, how WeWalk worked in practice and suggested changes. Interviews were audio-recorded on encrypted data recorders and transferred securely for professional transcribing.

### Data analysis

We collated descriptive data to characterise the sample. Interview data were analysed using thematic analysis [34]. We coded data using the Theoretical Framework for Acceptability (TFA) [35] which explores acceptability across domains described in Table 2. Dyads experiences of working together and of using intervention components were also coded inductively to explore their experiences. Interpretive themes relating to acceptability, experiences and walking behaviour were developed as analysis progressed. Two researchers independently coded interview transcripts (LI, TT) then agreed coding. We initially coded participant experiences of intervention components before examining the data through the lens of each of the TFA components, with overall positive responses on each construct as criteria for acceptability to support progression to trial. The outline coding tree is provided in Additional File 3. We developed our explanatory themes with a third researcher (JM). Data was managed using NVivo 12 software. We shared our interpretation with participants in an illustrated report that was posted to their home addresses and invited their feedback via email or telephone.

**Table 2:** Concepts within the Theoretical Framework for Acceptability [27] and their definitions

Concept	Definition
Affective Attitude	How an individual feels about WeWalk before, during or after taking part
Burden	The perceived amount of effort that is required to participate in WeWalk
Ethicality	The extent to which WeWalk has good fit with an individual’s value system
Intervention Coherence	The extent to which the participant understands WeWalk and how it works
Opportunity costs	The extent to which the benefits, profits or values must be given up to engage in WeWalk
Perceived effectiveness	The extent to which WeWalk is perceived as likely to or has achieved its intended purpose
Self-efficacy	The participant’s confidence that they can perform the behaviours required to participate in WeWalk

## Results

### Participant characteristics

Of 78 PWS approached, 21 (27%), 15 males and 6 females were recruited. Reasons for non-participation are presented in Table 3.

**Table 3:** Reason for not taking part

	**Number**
Refused, no reason given	11
No response received after initial invitation to participate	9
Health problems (stroke related), e.g. poor mobility, aphasia, fatigue, cognitive problems	7
Other health problems, e.g. Parkinson’s disease, bronchiectasis	7
Too many other commitments	6
Doing enough walking/exercise	6
Study stopped due toCOVID-19 restrictions before dyad could be recruited	5
Bad time of year (winter)	3
No walking buddy found before study was stopped due to COVID-19	2

We had anticipated recruiting 3–4 participants per month over 9 months. Actual recruitment rates at 3.5 ± 1.6 per month were as expected, until COVID-19 lockdown in March 2020 imposed by the United Kingdom Government, necessitated study cessation. At that point, ten dyads had received all intervention sessions, nine had completed some sessions and two had withdrawn due to ill-health and hospitalisation (Table 4). Table 4 shows recruitment from a range of settings was feasible, including exercise classes and stroke groups, although fewer were recruited through physiotherapy referral than anticipated.

**Table 4:** Recruitment sources, recruitment rates and number of weeks in the study (plus reason for non-completion)

Dyad recruited from	*N* = 21, *n* (%)
Exercise class	6 (29)
Referred by physiotherapist	7 (33)
Press release about study	3 (14)
Other (stroke club, PPI group, stroke charity, word of mouth)	5 (24)
Recruitment rate per month October 2019–March 2020	*N* = 21, *n* (%)
October	4 (19)
November	3 (14)
December	2(10)
January	5 (24)
February	6 (28)
March	1 (5)
Number of weeks in the study	*N* = 21, *n* (%)
12 weeks (i.e. completed all sessions before lockdown)	10 (50)
9 weeks (intervention stopped due to lockdown)	1 (5)
5–6 weeks (intervention stopped due to lockdown)	3 (15)
2–3 weeks (intervention stopped due to lockdown (4 dyads); withdrawn due to loss to follow-up (I dyad); the PWS had a hospital admission (2 dyads)	7 (30)

PWS were between 43 and 82 years of age (median 65.5 years) (Table 5). Time since stroke ranged from 1 month to 15 years, with 11 (50%) < 1-year post-stroke (Table 5). Seventeen lived with a spouse, partner, and/or family members, and four lived alone. All socio-economic categories were represented [36]. Baseline disability and frequency of walking varied (Table 5). Eight (38%) PWS seldom walked outdoors, while ten (47%) went out on most or all days. Rivermead Mobility Assessment Scale scores indicated a range of mobility levels, with 52% reporting good mobility, but PA levels were low with 81% of participants scoring < 110 on the Physical Activity Scale for the Elderly. Forty-eight percent of participants reported > 100 on the Activities Specific Balance Confidence Scale indicating good balance, but only 33% reported walking self-efficacy scores of > 20. Fatigue levels were > 21 l for all participants indicating high fatigue (Table 5).

**Table 5:** People with stroke: demography and baseline PA

Characteristic	*N* = 21, *n* (%)
Sex	
Male	15 (71)
Female	6 (29)
Living arrangements	
Lives with spouse, partner and/or other family members	17 (81)
Lives alone	4 (19)
Age	
< 55 years	5 (24)
55–65 years	7 (33)
> 65 years	9 (43)
Scottish Index of Multiple Deprivation (SIMD) decile	
1–3 (most disadvantaged)	7 (33)
4–7	6 (29)
8–10 (least disadvantaged)	8 (38)
Time since stroke	
< 1 year	11 (52)
1–5 years	6 (29)
> 5 years	4 (19)
Frequency of outdoor walking	
Never/seldom	8 (38)
2–3 times per week	3 (14)
Most days	7 (33)
Every day	3 (14)
Rivermead Mobility Index (RMI): minimum Score = 0, indicating poor mobility, maximum Score = 15, indicating good mobility	
< 10	5 (24)
10–12	5 (24)
13–15	11 (52)
PA Scale for the Elderly (PASE): minimum score = 0 indicating low activity, maximum score = 400 indicating high activity	
50–70	5 (24)
71–90	6 (29)
91–110	6 (29)
> 110	4 (19)
Activities Specific Balance Confidence Scale (ABC): minimum score = 0 indicating lowest balance confidence, maximum score = 160 indicating highest balance confidence	
< 50	4 (19)
50–100	7 (33)
> 100	10 (48)
Self-efficacy Scale for Walking: Minimum score = 7 indicating lowest self-efficacy for walking, Maximum score = 35 indicating highest self-efficacy for walking	
7–15	9 (43)
16–20	5 (24)
> 20	7 (33)
Fatigue Assessment Scale: Minimum score = 10 maximum score = 50) Scores 10–21 = no fatigue, Scores > 21 = substantial fatigue	
25–30	6 (29)
31–35	12 (57)
> 35	3 (14)

Characteristics of walking buddies are presented in Table 6.

**Table 6:** Walking buddies, composition of the dyads, and recruitment setting

Factor	*N* = 21, *n* (%)
Sex	
Male	2 (10)
Female	19 (90)
Age	
< 55 years	7 (33)
55–65 years	6 (29)
> 65 years	8 (38)
Walking buddy’s relationship to the PWS	
Spouse/partner	13 (62)
Other family member	4 (19)
Volunteer/support work/friend (unrelated)	4 (19)
Person with stoke and walking buddy live together	15 (71)

Despite COVID 19 restrictions, 18 dyads who had completed or partially completed WeWalk provided interview data face-to-face (*n* = 8) or by telephone (*n* = 10). Post-lockdown, we interviewed six cohabiting dyads using telephone speaker facilities and conducted individual telephone interviews with members of four non-cohabiting dyads (21 interviews total). Fifteen diaries were returned on study completion. Fourteen dyads completed or partially completed diaries, recording weekly goals, plans, step counts, times or places walked. Diary sections recording reflections on activity and progress were completed by 13 dyads, indicating adherence to plans. Diaries were completed by PWS (*n* = 6), walking buddies (*n* = 5) or both dyad members (*n* = 4). Three PWS chose to use apps or Fitbit to record steps. Eighteen PWS were provided with the pedometer, but for twelve it was inaccurate in recording steps. Two participants therefore switched to using time walked to monitor activity whilst other recognised the inaccuracy of the pedometers but continued to use them as indicators of step count.

### Qualitative findings

Dyads’ perceptions of acceptability and experiences of using We Walk were explored to identify refinements in preparation for future effectiveness testing, guided by the Theoretical Framework of Acceptability [35].

#### Theme 1: Acceptability evolves with experience

Participants were enthusiastic about WeWalk and understood how helpful dyadic working could be. We defined dyadic working as working together to identify goals, together planning how to achieve them, whilst developing strategies to cope with challenges that arose, and reviewing and reflecting on progress. Accountability to buddies and the social support provided by planning and enacting walking together were considered motivational. As participants experienced dyadic working, acceptability evolved further. Four subthemes linked to the TFA explain how dyads perceived acceptability: *give it time*, *effort rewarded*, *building confidence* and *one improvement leads to another.*

#### Theme 1: Sub-theme—give it time

For many PWS, WeWalk aligned with desire for independence and beliefs that their efforts would be rewarded. However, time was required to foster an understanding of WeWalk. Positive attitudes grew as participants tried and succeeded in using components of the intervention designed to support behaviour change, and achievements in walking were realised.

But now we are just, ‘Right we’ll go for a walk now’ whereas at the start, ‘Oh we’ve got to go for a walk, we’ve got to fit this in.’ (Laughs)...even like yesterday when it was raining and not very wonderful, we just went... (Buddy Dyad 06)

For many, WeWalk provided reasons to restart walking, and appreciation was strong when people had not walked much previously.

It kick started me in a new direction in life …I would never contemplate going for short walks before. Now I would make it part of daily living. (Male PWS, Dyad 17)

As familiarity with WeWalk grew, it prompted regular walking together, enhancing desire for improvement and engendering enjoyment of walking together.

In the past, we had been quickly out for a walk—I guess, We Walk was therapeutic and gave us permission to loiter and look at things... putting a structure on it could encourage you to do something instead of saying, “Well, I’ll maybe do it tomorrow”. (Male PWS, Dyad 15)

However, a few participants valued WeWalk less. Fieldnotes show these PWS engaged and progressed less than dyads whose perceptions of its coherence grew over time, coherence being the extent to which a participant understands We Walk and how it works, according to the theoretical framework of acceptability [35]. One female participant appreciated social interaction with her volunteer buddy but considered walking goals less important. Another male participant showed ambivalence because he felt he was improving anyway. These participants did not consider WeWalk components coherent, influencing their perceptions of acceptability.

I might have looked back [at the diary] and seen something I’d done at the start and think, “I’ll try that again and see how much I’ve improved.” But I think myself, I was improving every week with it anyway. To be honest it was more of a hindrance. (Male PWS, Dyad 10)

#### Theme 1: Sub-theme 2—effort rewarded

For most participants, the physical and psychosocial effort of engaging with WeWalk diminished as they overcame challenges and realised improvements. As they understood WeWalk and addressed challenges, participants viewed the effort as manageable, and concerns of burden eased.

Well, the first week I think he felt quite overwhelmed…but we thought I could write down everything in the diary and it worked quite well (Female Buddy, Dyad 16)

Physical challenges made walking effortful, including poor balance and walking ability, inclement weather, and unsafe environments. However, gradual improvements counterbalanced perceived burden. For a few participants with severe stroke, effort outweighed capacity to participate, resulting in limited engagement. Most dyads, however, drew on physical and psychological resources to address challenges.

I’m not frightened now of going more of a distance, or sometimes the pavements are a little bit rutted, I’ve just got to be careful, plan the route so I know where to go. (Male PWS, Dyad 07)

Most buddies were happy with their role as buddies, but some volunteer buddies were concerned about providing appropriate support. However, the burdens of caring decreased with careful facilitation, familiarity and realising physical and mental improvements for the PWS. For some buddies, accountability to WeWalk meant not having to nag the PWS to walk; for others, it meant no longer requiring a wheelchair to take the PWS to the supermarket or exercise class. Burden for buddies eased as responsibility for intervention processes, such as diary completion, transferred to the PWS and confidence in walking alone grew.

It’s taken a huge weight off me, I always felt quite responsible thinking you had to or should be doing a lot of walking … I think you’re much more confident, which is great, I’m not so worried now. (Female Buddy, Dyad 07).

#### Theme 1: Sub-theme—building confidence

Accomplishing goals restored lost autonomy to PWS, engendering self-efficacy for walking and supporting their engagement in other valued activities. Initial support from buddies with WeWalk components (planning, diaries, tracking progress) often increased confidence, agency and independence, sometimes dramatically.

Male PWS: I know when [my buddy] was there I felt confident, I didn’t feel I was going to fall.

Female Buddy: He even surprised me a few times and he said, “Oh, I just went to the shop last week on my own” and I was like, “Oh, really?” (Dyad 16)

However, goal achievement was difficult for a few PWS and could diminish self-efficacy. For others, structured reflection provided focus, enabling them to develop resilience despite setbacks.

We just said, ‘We didn’t have a good week last week but this week this is the one.’ Sometimes we managed it and sometimes we didn’t. But you can look back on it then which helps (Female Buddy, Dyad 06)

#### Theme 1: Sub-theme 3—one improvement leads to another

Many participants reported a range of inter-related improvements. Improved walking quality, stamina and balance facilitated psychological and social benefits, enjoyment and renewed confidence. This encouraged increases in and maintenance of outdoor walking, in turn sustaining motivation.

It did work because before I would have just thought, no, I’ll not bother today, I’ll do that tomorrow when it’s not raining or when it’s blowing a hooley… It’s been successful in that I have improved my walking; I walk more confident; I go further and longer, and I can do a reasonable pace now, so that has all improved. (Male PWS, Dyad 07)

Social benefits stemmed from enjoyment and renewed confidence, facilitating engagement in other activities, for example re-engaging with friends and neighbours. For some, instead of using a wheelchair or car, walking to significant places provided pleasure and restored old identities.

I always enjoyed going to supermarkets because of my work involvement with clients. But that had gone into abeyance. And I thought, well now, I can go and see interesting things and I’m walking all the time. (Male PWS, Dyad 5)

Participants perceived walking outdoors as life-enhancing, equating it to wellbeing and feeling more energetic—‘awake and alive’. Many walked in parks and the countryside fostering appreciation of nature. Walking in their communities facilitated social contact. For participants with mental health problems whose condition discouraged walking, being outdoors stimulated better mood and confidence. For them, walking outdoors generated a cyclical pattern of activity, better mood and sustained activity.

He says walking outside helps to clear his head. Makes it feel a bit better. Sometimes he feels down, and this helps. Also gives him the opportunity to meet people as well. (Fieldnotes, Dyad 8).

Thus, acceptability of WeWalk stemmed from understanding WeWalk and experiencing enjoyment and associated physical, psychological and social benefits. Although participation involved initial effort, realising benefits meant effort was rewarded.

Themes 2 and 3 examined dyad experiences in working with WeWalk.

#### Theme 2: Mutuality

Mutuality is defined as the tendency of relationship partners to think of themselves as members of a *dyadic relationship* rather than as distinct individuals [37]. It describes dyadic interdependence characterised by mutual engagement and accountability and underpinned by relational connectivity. Dyads who were mutually engaged were often, but not always, spousal dyads. They reported setting goals and planning achievements, agreeing on new challenges and places to walk together. They negotiated problems, such as poor weather and lack of time, and generated shared solutions. These qualities fostered shared enjoyment of walking and sustained intervention participation. For those dyads, WeWalk provided focus on being together and partnerships in walking.

It sounds like marriage guidance, but it has meant we talk to each other rather than just announcing to each other we’re doing this or that...it helps us care for each other I think, to have a consensus about what are we doing this week. (Female Buddy, Dyad 15)

Developing relational connectivity to create and sustain WeWalk was necessary where buddies were volunteers and relationships were new. Developing participation required careful facilitation and time. Buddies were concerned about doing the right thing and knowing how much the PWS could do. Some PWS feared burdening buddies and sought assurance that buddies would also benefit. However, reciprocity evolved through thoughtful facilitation, planning and mutual understanding, ensuring both parties could work with WeWalk.

We just took it slowly and chatted ... it wasn’t the best first walk, but it was enlightening ... I benefitted from [the facilitator] doing goals and plans to see how to elicit information and what counts towards a goal. (Female Buddy, Dyad 20)

However, a few relationships, including spousal dyads, showed less mutuality and reciprocity, making negotiating goals and planning difficult. Those dyads showed low interaction with study materials, diary entries were scant and they reported less progress.

Facilitator: the PWS makes up his mind to do things and won’t be shifted. Buddy (wife) thinks he will not always listen to her. (Fieldnotes, Dyad 05). Buddy: Whether what I say will have any bearing on what he decides as his target is quite a different matter (Female Buddy, Dyad 05)

In summary, mutuality and reciprocity were central to the success of we walk, characterised by sharing decisions and working together to plan and achieve goals.

#### Theme 3: Person-centredness with personally relevant tailoring

Findings indicate that person-centred adaptability of WeWalk components was valued and considered essential for We Walk participation in this heterogeneous stroke population. Person-centredness in delivery was a fundamental principle of WeWalk and was enacted by enabling dyads to set their own goals and to use and tailor intervention components in ways that matched their values, circumstances and progress. These adaptations were relevant to individuals, whilst maintaining the integrity of theoretically driven components of WeWalk. This contrasted with the more directive approaches to exercise promotion that participants had experienced when working with health professionals. Consequently, dyads valued the ownership of goal-setting that WeWalk gave them,in together negotiating personal, achievable goals and planning how to enact them.

You have got to have structure and set goals in the first place, and we were trying to set goals as we are going along and say, ‘Right another five minutes or another ten minutes’ and such like. (Male PWS, Dyad 06).

PWS: So, you put no pressure on them to do it, you say, there it is, just tell us what is important to you to do. Just let them get on with it (Male PWS Dyad 09).

Facilitator: So, you think that was the right approach?

PWS: Oh, aye, without a doubt. I think if you push somebody into something it means, I’ve got to dae it if you’re that way inclined, you know—I want to do it; you want to do it or you’ve got to do it, this is two different things (PWS Dyad 09)

Field notes reflected dyads’ desire to set and achieve goals immediately. Goal setting was originally incorporated into the second intervention session, as baseline assessment and explanation of dyadic working and setting up pedometers took up most of session one. However, participants’ desire to start setting initial goals immediately led us to change the delivery plan to integrate all these components into the first session to accommodate their enthusiasm. Participants adapted walking goals and plans depending on progress and their immediate context. Some adjusted walking around how they felt on a given day. Others adapted by using treadmill walking in inclement weather or created challenges by walking further, faster or to new places.

The better he was getting, it was harder to say a further goal, but it was a case of improving and going as far and as quick as you could; just improving all the time. (Female Buddy, Dyad 08).

Recording and monitoring progress was a motivational intervention component that facilitated review and appraisal. Many found pedometers helpful; however, slow walking was often not captured, leading to a dissonance between actual and registered steps and disappointment. However, participants adapted by recording time or distance walked, using mobile phone apps or activity trackers to monitor progress.

We were using time as a guide to not overdo things. You said, that is 12 or 13 min ‘How are you feeling?’ Do a wee bit more and then, we have got to get back. (Male PWS, Dyad 06)

In summary, the qualitative data showed that WeWalk was acceptable to dyads and was influenced by growing familiarity with We Walk. The physical benefits, enjoyment and growth in confidence that it provided to the PWS meant that initial effort of participation receded. Engagement was influenced by how well dyads worked together, with those exhibiting mutuality participating most successfully. The heterogeneous nature of the stroke population meant that person-centred adaptability to the persons’ context, physical capabilities, values and personal goals was important to PWS and facilitated their engagement with WeWalk. Integration of goal setting into session one from session two was a necessary refinement. Development of more specific strategies to train buddies in how best to support PWS to walk is another necessary development indicated by the data.

## Discussion

Recruiting dyads to WeWalk was feasible, and qualitative data shows participants regarded WeWalk as acceptable. Mutual engagement of dyads facilitated participation in WeWalk leading to physical, psychological and social benefits. Person-centred adaptation of WeWalk to individuals’ contexts and disabilities facilitated participation whilst preserving core theory-informed components known to promote behaviour change. As well as recruiting buddies known to people with stroke,  we recruited volunteer buddies from local charitable organisations. Prior to subsequent studies, generation of partnerships with stroke charities and careful training of volunteers to ensure sustainable recruitment of volunteers and implementation will be important.

Until disrupted by COVID-19 lockdown, we recruited on average 3.5 dyads per month as initially anticipated, indicating recruitment was feasible, but recruiting fewer participants via physiotherapy than community routes, was not anticipated. Physiotherapists identified potential participants; however, it is likely that they acted as gatekeepers to recruitment, proposing PWS they thought could participate, thereby leading to some selection bias. For future research, we would include clinical research staff to support recruitment and undertake more education work with physiotherapists to ensure they give all PWS who were able to walk the option of taking part. Furthermore, healthcare commitments and adjusting to life with stroke often made participation difficult. Recruiting participants later in their recovery when rehabilitation ends may be more appropriate. However, participants may become deconditioned making later intervention engagement difficult, requiring careful progression of walking to match their capabilities. Although most buddies were spouses, volunteer buddies were recruited and participated, confirming that implementation in different contexts is feasible with careful facilitation. Participants were diverse in disability, baseline activity and socio-economic status. Despite the heterogeneity, engagement with We Walk components was high.

The Theoretical Framework for Acceptability (TFA) [35] guided qualitative data interrogation. The concept of coherence reflects peoples’ understanding of interventions and how they work—reflecting an individual’s understanding of the perceived level of ‘fit’ between the components of the intervention and the intended aim of the intervention [35]. Experiencing WeWalk ‘in use’ enhanced participants’ appreciation of dyadic working, supported by careful facilitation. Consequently, their appreciation of the coherence of WeWalk components in supporting walking grew. Guided by data, we incorporated goal setting into session one but found little evidence suggesting further refinements to enhance coherence.

Ethicality within TFA reflects intervention alignment with participants’ values. Data suggests tailoring WeWalk according to person-centred principles placed individual participant values as central to their decision-making and experiences. The willingness of buddies to support PWS, along with dyads’ expectation of benefits for PWS of working together, reflects the concept of ethicality. Also aligned with ethicality, WeWalk engaged participants’ social support structures, seeming to work best with mutually engaged, often spousal, dyads. Findings concur with a randomised controlled trial of a dyadic intervention to promote PA with community-dwelling couples [38], which found dyads with low relationship quality, as measured by questionnaire, demonstrated lower PA levels for the target person. As with WeWalk, less connected dyads differed in planning and goal enaction ideas and reported fewer benefits.

Diary entries suggest that the dyads demonstrating mutual engagement with WeWalk engaged most with planning and seeking to achieve goals. Findings suggest successful engagement with WeWalk rests with qualities of dyadic relationships and buddies’ support skills. Assessing these qualities prior to WeWalk participation will in future guide delivery of targeted training, and assist buddies within existing and new dyads in providing appropriate support. Processes will be included in subsequent WeWalk research. Dyads with volunteer buddies worked well, bypassing complex relationship challenges, although matching and developing these dyads required time and careful facilitation. Other walking intervention studies have successfully used volunteer buddies [39], and the option should be pursued more extensively for the next WeWalk study phase.

Our person-centred approach reflected the research team’s ethical stance, aligning with TFA ethicality. Person-centredness resonated with participants, supporting their desire for autonomy [26], and facilitating meaningful engagement for dyads, whilst retaining the overall purpose of WeWalk and its theoretical basis in behaviour change. Personal tailoring of some intervention components enabled participants to engage despite their disabilities, for example, finding ways to monitor progress when, as other studies have illustrated, pedometers did not register slow walking [40]. Congruently, a qualitative meta-synthesis [41] of people with disability highlighted desire for autonomy in engaging in PA. Another qualitative study [42] showed PWS desire to take responsibility for their health and valued diverse opportunities for accessible community exercise. Together, the studies endorse our operationalisation of person-centredness in WeWalk, through tailoring to participants’ contexts and values.

Our person-centred approach differs from prescriptive approaches to PA within evidence-based stroke guidelines [1, 2]. The approach we adopted to increasing walking behaviour in meaningful, enjoyable ways, embedded in everyday life and tailored to individual capabilities should however create long-term habits that support PWS to maintain PA levels recommended in guidelines, and develop confidence for other types of PA [10].

During WeWalk development, PWS emphasised the importance of outdoor rather than indoor walking because being outdoors improved wellbeing and was, for many, a recovery goal [21]. Congruently, data shows WeWalk participants experienced enhanced confidence, wellbeing, social connection and alertness from being outdoors. A qualitative study by Stretton et al. [13] showed PWS perceived improved confidence, motivation, social support and physical and psychological outcomes through outdoor walking. Findings align with public health research that being outdoors and exposure to green space reduces stress, anxiety, depression, and mental fatigue [43, 44]. Focusing on outdoor walking in WeWalk thus addressed a fundamental human need essential to all.

Despite initial concerns, physical, social and psychological benefits of participation offset any burden of participation for most dyads. Furthermore, participants reported perceived effectiveness characterised by enhanced wellbeing, social interaction and independence. Effects were underpinned by increased enjoyment of walking, often for both dyad members. Another construct of acceptability, self-efficacy, confidence to perform behaviours required to participate in WeWalk, grew through participation. As people experienced benefits of WeWalk, most reported enhanced confidence in using We Walk and in walking itself. WeWalk reflects the growing importance of behavioural interventions for PA promotion in stroke [45], building on earlier interventions involving exercise instructions alone that were not effective in promoting long-term PA participation [46]. Components included in WeWalk align with those within a review of behavioural strategies for PA promotion after stroke [15], indicating consensus is growing about intervention characteristics for PA promotion after stroke. Further evaluation of optimal intervention components and behaviour change strategies is required, given that interventions incorporating these strategies are more likely to increase long-term PA participation after stroke more than directed exercise alone [10, 15].

### Strengths and limitations

This novel study explores a dyadic intervention for PA promotion after stroke and represents a feasible and acceptable way to support outdoor walking as PA. We adapted WeWalk to enable participants to start setting goals immediately, but other WeWalk elements did not require refinement. Before testing effectiveness, we will explore optimal routes and timing for community recruitment, given lower rates of recruitment through physiotherapy than anticipated. We will also explore ways to examine dyadic relationships and the support skills of buddies for WeWalk, to implement training in provision of support when indicated. Although we explored how WeWalk worked to determine how best to refine it, this stage of intervention development did not evaluate effectiveness. Furthermore, we did not set specific criteria for progression to randomised controlled trial. However, recruitment of dyads was feasible and qualitative acceptability data suggest progression is appropriate. COVID-19 lockdown meant intervention completion was only possible for some participants, inevitably influencing the perceptions expressed in interviews. The sample was predominantly male. In the next stage of this research, we will specifically target recruitment of more female participants to ensure generalisability. The interview researcher also delivered WeWalk, potentially causing social desirability bias in interviews. However, participants’ views were not universally positive, suggesting they were comfortable providing honest accounts.

## Conclusions

WeWalk is a novel dyadic intervention to support walking as PA after stroke. Notwithstanding COVID-19 lockdown, recruiting and retaining dyads was feasible. WeWalk was acceptable to participants and perceptions of acceptability grew as dyad members realised benefits. WeWalk worked best in mutually engaged dyads; therefore, evaluating relationships and developing support skills for buddies will be important for the next stage of WeWalk research. This study provided important information on intervention delivery and recruitment aspects to be refined before effectiveness testing. Community recruitment and delivery strategies need to be developed next, in collaboration with relevant partners.

## Supplementary Information

**Additional file 1.** Baseline assessment measures. Measures used to describe characteristics of people with stroke at baseline.

**Additional file 2.** Interview guide.

**Additional file 3.** Qualitative data coding tree. The TIDieR framework.

## Data Availability

The datasets used analysed during the current study are available from the corresponding author on reasonable request.

## Abbreviations

PA: Physical activity
PWS: People with stroke
TFA: Theoretical framework of acceptability
