# Supplementary material for: WeWalk: walking with a buddy after stroke—a pilot study evaluating feasibility and acceptability of a person-centred dyadic behaviour change intervention
Source: Pilot Feasibility Stud. 2023 Jan 13;9:10. doi: 10.1186/s40814-022-01227-5 (PMC9837756; doi:10.1186/s40814-022-01227-5)
Supplement: Supplementary file 1 — Additional file 1. Baseline assessment measures. Measures used to describe characteristics of people with stroke at baseline. [file 40814_2022_1227_MOESM1_ESM.docx]

**Additional File 1**

*.dox

Baseline Assessment Measures

Measures used to describe characteristics of people with stroke at baseline.

**The Physical Activity Scale for the Elderly (PASE) (1)**

The PASE is a validated 12-item self-administered questionnaire that measures the amount of physical activity undertaken. The scale assesses the types of activities typically chosen by older adults (walking, recreational activities, exercise, housework, yard work, and caring for others [12]. It uses frequency, duration, and intensity level of activity over the previous week to assign a score ranging from 0 to 400, with higher scores indicating greater physical activity. It has been widely used to evaluate self-reported physical activity after stroke and reflects physical capacity to perform everyday activities that require strength, aerobic endurance and balance in persons suffering from stroke (1).

**Rivermead Mobility Index (2)**

The Rivermead Mobility Assessment is a 15 item self-assessment questionnaire that assesses functional mobility following stroke. Items are coded as 0 or 1 depending on whether the person with stroke can complete the task according to specific instructions. Total score is determined by summing the points for all items. The maximum score is 15 indicating better mobility performance. The scale is extensively used in stroke research and validity and reliability are established (2, 3).

**The Activities Specific Balance Scale (3)**

The Activities Specific Balance Confidence Scale is a 16-item self-report measure in which patients rate their balance confidence in performing various activities. Items are rated on a scale ranging from 0-100 in which a score of 0 represents no confidence and a score of 100 represents complete confidence. The scale has excellent test-retest reliability in chronic stroke and correlates with other measures of balance used in that population (4).

**Self-Efficacy Scale for Walking (4)**

The Self-Efficacy for Walking Scale is a self-report scale examining self-efficacy for walking in people with ischemic stroke and transient ischemic attack (5). The total score of the SEW-7 ranges from 7 to 35 points with higher scores indicating better self-efficacy. The scale has good internal consistency and test-retest reliability.

**Fatigue Assessment Scale (5)**

The FAS is a 10-item scale evaluating symptoms of chronic fatigue that is valid and reliable in stroke (6). The scale is a 10 item scale Each item of the FAS is answered using a five-point, Likert-type scale ranging from 1 (“never”) to 5 (“always”). Items 4 and 10 are reverse scored. Total scores can range from 10, indicating the lowest level of fatigue, to 50, denoting the highest.

References

1. Washburn RA, Smith KW, Jette AM, Janney CA. The physical activity scale for the elderly (PASE): Development and evaluation. Journal of Clinical Epidemiology. 1993;46(2):153-62.
2. Collen FM, Wade DT, Robb GF, Bradshaw CM. The Rivermead Mobility Index: A further development of the Rivermead Motor Assessment. International disability studies. 1991;13(2):50-4.
3. Botner EM, Miller WC, Eng JJ. Measurement properties of the Activities-specific Balance Confidence Scale among individuals with stroke. Disability and Rehabilitation. 2005;27(4):156-63.
4. Kawajiri H, Adachi T, Kono Y, Yamada S. Development of a Self-Efficacy Questionnaire for Walking in Patients with Mild Ischemic Stroke. J Stroke Cerebrovasc Dis. 2019;28(2):317-24.
5. Michielsen HJ, De Vries J, Van Heck GL. Psychometric qualities of a brief self-rated fatigue measure: The Fatigue Assessment Scale. Journal of Psychosomatic Research. 2003;54(4):345-52.
