# Supplementary material for: WeWalk: walking with a buddy after stroke—a pilot study evaluating feasibility and acceptability of a person-centred dyadic behaviour change intervention
Source: Pilot Feasibility Stud. 2023 Jan 13;9:10. doi: 10.1186/s40814-022-01227-5 (PMC9837756; doi:10.1186/s40814-022-01227-5)
Supplement: Supplementary file 2 — Additional file 2. Interview guide. [file 40814_2022_1227_MOESM2_ESM.docx]

**Additional File 2.** *.dox

**Interview Guide**

The researcher conducted a face-to-face or telephone interview with both members of the dyad at the end of WeWalk delivery period. If the participants stopped WeWalk because of COVID lockdown or for other reasons, the interview was conducted at that time. Topics explored through discussion were:

**Working as a dyad**

- How dyadic planning worked
- Frequency
- Planning process
- Walking together or not
- How did the dyad feel about undertaking tasks such as goal setting, self-monitoring?
- Were these tasks manageable and helpful?
- Which strategies used did they prefer
- How did the dyad cope with challenges and how did they come up with solutions?
- If the person with stroke successfully increased walking outdoors, how did working with a buddy help to achieve that?
- How could the research team have helped to facilitate dyadic working?

**Monitoring walking**

- How easy was it to use pedometers or other devices to monitor walking?
- Were these devices helpful in increasing motivation to walk? If so, how?
- What information recorded in the diaries was useful in monitoring progress?
- What other information would you want to record and how often?
- Would alternative formats for keeping a diary e.g., voice recording rather than written be more useful?
- How did you complete the diary (together, person with stroke only, buddy)?
- Would it be helpful if both members of the dyad kept a diary?
- Benefits and challenges of walking
- In general, what was your experience of taking part in the study/intervention
- worthwhile/enjoyable/helpful/difficult/challenging (individually and as a dyad)?
- What benefits, if any, were there from walking more? (Explore physical, social, and psychological benefits).
- What were the challenges of walking more? (Explore personal, social, environmental)
- How did you overcome the challenges?

**Support from the research team**

- How did you view the support you received from the study (individually and as a dyad)?
- What other support would be helpful?
- As a dyad, how did the way the programme was delivered enable you to tailor WeWalk and come up with options for walking that suited your needs and wants?

**Acceptability of the study**

- As a dyad, what did you think about the duration of WeWalk?
- Would you have liked it longer or shorter?
- Would you have liked contact?
- What would be the optimum number of face-to-face contacts?
- How can WeWalk be improved?
- Who would be the best person to deliver WeWalk when it is rolled out?
- When should WeWalk be delivered?
- In what ways do you think the handbook could be improved?

**About taking part in the study**

- How did you feel about being approached to take part in the study?
- What do you remember most about the sessions?
- Views on home visits
- Views on telephone contacts
- Did you discuss the study with anyone apart from your walking buddy? If yes, with whom
- Would you recommend the study to anyone? If yes, whom?
