# Supplementary material for: WeWalk: walking with a buddy after stroke—a pilot study evaluating feasibility and acceptability of a person-centred dyadic behaviour change intervention
Source: Pilot Feasibility Stud. 2023 Jan 13;9:10. doi: 10.1186/s40814-022-01227-5 (PMC9837756; doi:10.1186/s40814-022-01227-5)
Supplement: Supplementary file 3 — Additional file 3. Qualitative data coding tree. The TIDieR framework. [file 40814_2022_1227_MOESM3_ESM.docx]

**Additional File 3.** *.dox

**Qualitative Data Coding Tree**

**Coding for Intervention Components**

Action planning

Buddy

- Benefits
- Burden
- Dyadic Planning
- Role
- Support
- Who

Diary

Dyad desired outcomes

Dyad learning and adjustment

Goals

Handbooks

Intervention delivery

- Changes (recommended by Dyad)
- Duration of the intervention
- General comments
- Number of contacts in the intervention
- When to deliver (post-stroke)

Knowledge and understanding

Maintenance

Motivation

Outcomes

Outside or Outdoors

Pedometers and apps

Person-centred processes

Resources available

Role of the implementer

Stroke survivors' experience of stroke

**Coding for the Theoretical Framework of Acceptability (TFA)**

**TFA Affective attitude**

- Pleased to be invited to take part
- Told others about the study (and benefits), recommend it for others
- Coherence of the intervention encouraged participation
- Anticipated benefits (prospective)
- Experiencing benefits (concurrent)
- Experienced benefits (retrospective)
- Altruism/commitment to the study
- Understanding the benefits, the intervention can bring
- Credible source/Role of facilitator

Feelings about walking

- Change in attitude about walking
- New hobby
- Knowledge to experience changed their attitudes
- "Positive Belief in the potential benefits of the intervention
- Change in affective attitude (about the study) over time with experience.
- Emotions changed through experience of walking, which supported the change in their behaviours (back to golf)"
- Knew benefits but didn’t do it, "why can’t we do it ourselves?) Enjoyment of walking (changed behaviour) and of taking part discovery of enjoyment(fieldnotes)
- Enjoyment of walking (changed behaviour) and of taking part
- Richer understanding of value and pleasure of walking

**TFA Burden**

- SS doesn’t always listen to the buddy
- Inclement weather, particularly wind and ice
- Lack of time to fit in around other commitments
- Keeping the diary
- Commitment to the study
- Cognitive problems
- Feeling overwhelmed
- Walking e.g., in a supermarket, takes longer with the SS
- Low mood
- Lack of access and opportunities for walking
- Buddy commitments e.g., work
- Health problems
- Duration of the intervention
- Built environment e.g., traffic light, kerbs, pavements, terrain
- Embarrassment
- Feeling a burden to the buddy
- Living alone

**TFA Ethicality**

- Study from a credible source
- Exercise is important for health
- Enjoyment of walking and the outdoors
- Value doing things together
- Valuing work, but changing perspective on what constitutes work i.e., seeing the intervention as work to aid recovery
- Determination, willingness to work to make progress
- Altruism
- Feeling an equal among peers (and with volunteer buddy)
- Honesty about the utility of the intervention
- Green values)
- Reciprocity
- Having structure in your day (avoid procrastination)
- Being valued as a person
- Not being a burden (negative)
- Respect/mutual understanding (volunteer stroke survivor as buddy)
- Being independent

**TFA Intervention coherence**

- Coherence of the whole intervention
- Self-monitoring
- Looking for opportunities for walking in daily life
- Enjoying being out together
- Action planning (long-term)
- Dyadic working
- Diary for accountability
- Learning how to be a good walking buddy
- Structure for walking
- Make explicit what we want to achieve
- How the study increased motivation

**TFA Opportunity costs**

- Time
- Other commitments
- Access to equipment, wet weather gear
- Neglecting other exercises

**TFA Perceived effectiveness**

*Satisfaction*

- Overall satisfaction
- Enjoyment in the dyad doing things together (coded separately)
- Recommend it for others
- Increased confidence (coded separately)
- Increased motivation (coded separately)
- Potential benefits of the intervention to other people

*Physical benefits*

- Seeing progress with walking
- Feeling better (general)
- Increased energy/stamina
- Balance
- Coping with pain
- Progress in other activities and areas of life
- Seeking new opportunities for walking
- Walking becoming part of daily routine

*Psychological/social benefits*

- Mental health
- Increased social contact
- Helps with cognitive problems
- Other people noticing improvement
- Acceptance of stroke

**TFA Self-efficacy**

**What increases self-efficacy**

- Getting back to previous activities i.e., pre-stroke
- Overcoming challenges/coping planning e.g., poor weather/difficult terrain
- Achieving goals/seeing progress in walking
- Achieving/gaining confidence in other activities
- Looking for opportunities to walk more/do other things
- Changing expectations and ambitions (acceptance of stroke)
- Altruism
- Getting out of the wheelchair, talking to people at eye level
- Getting started/challenging yourself to walk more
- Study ‘giving permission’ to walk more
- Working together to achieve things
- Support from study
- Pre-stroke experience/ability
- Building up walking gradually
- Finding a safe place to walk
- Seeing improvements in others
- Support from other people e.g., friends, neighbours
- More mentally active through addressing challenges
- Enjoyment
- Setting achievable goals

**What is unhelpful for self-efficacy?**

- Timing is wrong, unable to give the study enough attention
- Not achieving goals (not We Walk related)
- Inability to focus (cognitive problems)
- Low mood
- Poor weather (particularly wind)/difficult terrain
- Poor balance/fear of falling
- Being with other people when confidence is low
- Afraid of taking the first step i.e., going outside to walk
- Non-acceptance of the stroke i.e., thinking it will get better (like a broken leg)
- Lack of opportunities to get out
- Thinking you can’t do it

**Benefits of increased confidence**

- Progress in other activities
- Use stick less
- Able to walk alone
- Took over writing the diary Explain to dyads that each other’s role in We Walk can change
- Takes burden away from the buddy
- Not having to watch your feet all the time
- Being able to hold a conversation when walking

**Additional File 3 .dox***

| **The TIDieR Framework** | |  |
| --- | --- | --- |
|  | Page of manuscript | Notes |
| Name or phrase to describe intervention | 7 |  |
| Describe any rationale, theory, or goal of the elements essential to the intervention | 8-9 |  |
| Describe any physical or informational materials used in the intervention, including those provided to participants or used in intervention delivery or in training of intervention providers. | 9 | We have not provided the materials in full because the intervention has not been tested in a randomised controlled trial yet and we need to preserve it as our intellectual property until then. |
| Describe each of the procedures, activities, and/or processes used in the intervention, including any enabling or support activities | 9-11 |  |
| For each category of intervention provider (for example, psychologist, nursing assistant), describe their expertise, background and any specific training given | 9 |  |
| Describe the modes of delivery (such as face to face or by some other mechanism, such as internet or telephone) of the intervention and whether it was provided individually or in a group | 9-10 |  |
| Describe the type(s) of location(s) where the intervention occurred, including any necessary infrastructure or relevant features | 9 |  |
| Describe the number of times the intervention was delivered and over what period of time including the number of sessions, their schedule, and their duration, intensity or dose | 9-10 |  |
| If the intervention was planned to be personalised, titrated or adapted, then describe what, why, when, and how If the intervention was modified during the course of the study, describe the changes (what, why, when, and how) | 24 |  |
| If intervention adherence or fidelity was assessed, describe how and by whom, and if any strategies were used to maintain or improve fidelity, describe them | 9 and 10 |  |
| If intervention adherence or fidelity was assessed, describe the extent to which the intervention was delivered as planned | 10 | We recorded detailed fieldnotes to capture delivery and participant engagement with We Walk and to identify any required refinements. Adherence was examined by diary completion by particpants |
